# The Use of Intravenous Vitamin C as a Supportive Therapy for a Patient with Glioblastoma Multiforme

**DOI:** 10.3390/antiox7090115

**Published:** 2018-08-30

**Authors:** Nicola Baillie, Anitra C. Carr, Selene Peng

**Affiliations:** 1 Integrated Health Options Ltd., Auckland 1050, New Zealand; 2 Department of Pathology & Biomedical Science, University of Otago, Christchurch 8140, New Zealand; anitra.carr@otago.ac.nz; 3 Feedback Research Ltd., Auckland 1050, New Zealand; selene@feedbackresearch.co.nz

**Keywords:** vitamin C, intravenous vitamin C, ascorbic acid, glioblastoma, neoplasms, quality of life

## Abstract

Glioblastoma multiforme is a high grade malignant brain tumour with a poor prognosis. Here we report the case of a woman with glioblastoma who lived for over four years from diagnosis (median survival 12 months and 2% survival for three years), experiencing good quality of life for most of that time. She underwent initial debulking craniotomy, radiotherapy and chemotherapy, as well as having intravenous vitamin C infusions 2–3 times weekly over the four years from diagnosis. Her progress was monitored by blood tests, regular computerised tomography (CT) and magnetic resonance imaging (MRI) scans, clinical reviews and European Organization for the Research and Treatment of Cancer quality of life questionnaires (EORTC QLQ C30). Our case report highlights the benefits of intravenous vitamin C as a supportive therapy for patients with glioblastoma.

## 1. Introduction

Glioblastoma multiforme is a high grade malignant brain tumour with a poor prognosis; median survival 12 months and 2% survive for three years [1,2]. We describe the case of a 55-year-old woman who responded much better than expected, with good quality of life for almost four years from diagnosis. She chose to combine conventional treatments with intravenous (IV) vitamin C therapy. Although there are limited clinical trials on the use of IV vitamin C for people with cancer, the available evidence indicates it is safe and generally well tolerated when combined with standard cancer therapies [3,4,5]. A small trial of IV vitamin C in combination with standard therapy in glioblastoma patients indicated a trend towards enhanced median survival [6]. In our case, the IV vitamin C was well tolerated, quality of life improved markedly in the first year, and then stabilised, and the patient had improved or stable blood tests throughout, including normal renal function.

## 2. Case Report

The enduring power of attorney provided consent for the presentation of the patient’s case. The 55 years old woman was diagnosed in November 2010 with glioblastoma multiforme following a 10 days history of headaches and constipation. Concurrently, she was diagnosed with extranodal marginal zone lymphoma, affecting the left parotid gland and bladder. She was previously fit and healthy, with no significant past medical history, and no history of renal stones. She did not drink alcohol and was a nonsmoker. Her sister died of a brain tumour age 40, and her father of prostate and bladder cancer age 84. She underwent craniotomy and debulking surgery in December 2010, followed by a course of radiotherapy and temozolomide. She had no specific conventional treatment for the lymphoma. 

Overall, the patient received 25 radiation treatments March to April 2011, and a further 10 treatments April 2014, and temozolomide for six cycles from March to June 2011, and a further six cycles from April 2014. She had no conventional cancer treatments for almost three years, from July 2011 until April 2014.

### 2.1. IV Vitamin C Treatment

When the patient first presented to our clinic mid-January 2011 she had fatigue, lethargy and an early chest infection. She was diagnosed with pneumonia the following day, which was treated with IV antibiotics in hospital, at which time she also had a blood transfusion for anaemia. The patient commenced regular IV vitamin C infusions a few days later, approximately three weeks following her brain surgery for glioblastoma multiforme. Baseline blood tests showed normal renal function and normal glucose-6-phosphate dehydrogenase (G6PD) status. If G6PD deficiency is present we do not recommend more than 25 grams IV vitamin C as there is an increased risk of haemolysis. Her non-fasting baseline plasma vitamin C level was 1.2 mg/dL (68 µmol/L); she had consumed a 1 g vitamin C supplement prior to coming to the clinic. The patient received IV vitamin C working up to 85 g/infusion, three times/week for the first six months to June 2011, then twice/week for over three years, stopping October 2014. Post-infusion plasma vitamin C levels were monitored for the first four months after she commenced IV vitamin C infusion to ensure that the proposed ascorbic acid therapeutic level was achieved. The average post-infusion plasma vitamin C level was 393 mg/dL (22 mmol/L), with a dose of 85 g (1.1 g/kg). The patient’s renal function tests were normal throughout IV vitamin C treatments with creatinine ranging from 51–67 µmol/L and eGFR mostly >90 mL/min/1.73 m^2^. The IV vitamin C was well tolerated.

### 2.2. Outcome and Follow-Up

CT scans or MRI scans with contrast were carried out every 3–6 months over the four years from diagnosis. These indicated stable disease until March 2014 (Table 1). In July 2011, her extranodal marginal zone lymphoma, eight months after diagnosis with no specific lymphoma treatments, showed stable disease on CT scan of the left parotid gland, with a possible decrease in the size of bladder mass. Six monthly follow-ups reported stable disease until March 2014 when a MRI scan noted lymphoma increase in both orbits, but the comment was made that ‘overall lymphoma was reasonably stable’.

Following the initiation of IV vitamin C treatments (January 2011), the patient reported clinical improvements that were maintained for almost four years following diagnosis. She noticed improved energy and walking distance four weeks after initially commencing IV vitamin C, prior to starting her first course of radiation therapy. She chose to continue IV vitamin C throughout her two courses of chemotherapy and radiotherapy, despite being advised that we do not recommend IV vitamin C concurrently with these treatments as there is limited research into benefits or interactions in this area. She felt it helped her symptoms and reported tolerating the radiotherapy ‘pretty well’.

The patient’s health-related quality of life (QoL) was monitored using the EORTC QLQ C30 for the first 12 months, and subsequently through clinical reviews. Compared to the patient’s QoL data prior to commencing IV vitamin C treatment, dramatic improvement was reported in the three months survey following initiation of IV vitamin C treatment (at that time the patient had just completed radiotherapy and started chemotherapy) and the improvement was maintained for the first 12 months (Table 2). Clinical symptoms such as fatigue, dyspnoea, insomnia, appetite loss and diarrhoea resolved and the patient’s physical and role functioning increased significantly, as well as ‘global health status’, which improved from ‘very poor’ to ‘excellent’. 

Formal QoL monitoring was not collected after the first 12 months, but from clinical reviews the patient maintained a good QoL, feeling ‘very well’ until April 2014 when she developed seizures. These were well controlled with medication and she continued working full-time until October 2014. Early November she deteriorated with reduced alertness, right sided weakness, and expressive dysphasia. She ceased IV vitamin C at this time. She continued to deteriorate until 26 February 2015 when she passed away at home, four years and three months from the initial diagnosis.

## 3. Discussion

The use of pharmacological ascorbate as an adjuvant cancer therapy was proposed as early as 1976 [7]. More recent studies have consistently shown selective toxicity to cancer cells compared to normal cells in vitro and in vivo [6,8,9,10,11]. The mechanism of action for selective cancer cell toxicity is not fully understood, although several research studies have suggested that the cytotoxic effect is mediated through ascorbate-mediated reduction of catalytic metal ions and subsequent generation of hydrogen peroxide, which can induce oxidative damage to macromolecules (i.e., DNA, proteins and lipids), deplete cellular adenosine triphosphate (ATP), and thereby cause cell death [12,13,14]. Another plausible mechanism of action has been recently proposed, as high dose ascorbate administration has been shown to slow tumour growth and decrease hypoxia through suppressing the activity of hypoxia-inducible factor (HIF-1), which is known to contribute to tumour progression [11,15,16]. Clinical trials in the use of IV vitamin C in critically ill patients [17,18,19] and patients with cancer [3,4,5,20,21] have demonstrated lack of toxicity, good safety and tolerability [22].

Our case report outlines the unexpected increase in progression free and overall survival in a woman with glioblastoma who had IV vitamin C from shortly after diagnosis, for almost four years, stopping three months before she died. She chose to continue IV vitamin C throughout her two courses of chemotherapy and radiotherapy and her increased survival time supports the findings of a small Phase 1 clinical trial in the concurrent use of IV vitamin C in patients with glioblastoma receiving chemotherapy and radiotherapy [6]. Although the IV vitamin C was the only additional treatment she was undertaking we cannot conclude that it was the primary factor in her increased progression free survival. However, it is interesting to note that epigenetic dysregulation of the vitamin C-dependent DNA hydroxylase ten-eleven translocation-2 (TET2) is observed in human glioblastoma and decreased hydroxymethylcytosine is associated with shorter malignant glioma survival [23,24], suggesting a possible mechanism by which vitamin C could aid in glioblastoma therapy. Nevertheless, it is likely that the IV vitamin C contributed to the patient’s improved quality of life, as has been observed in numerous other studies [25]. 

## 4. Conclusions

Our case report indicates that IV vitamin C may be a useful supportive therapy in the management of people with glioblastoma multiforme. IV vitamin C even at doses of over 1 g/kg body weight was very well tolerated. There were no renal stones or impairment of renal function throughout four years of treatment with IV vitamin C in this case. Overall, IV vitamin C given concurrently with chemotherapy and radiotherapy had no apparent adverse reactions and contributed to an improved quality of life and potentially an increase in progression free and overall survival. Further trials on the use of IV vitamin C as an adjuvant therapy in people with glioblastoma are warranted. Currently there is a Phase II trial underway investigating pharmacological vitamin C combined with radiation and temozolomide in glioblastoma multiforme (NCT02344355).

## Figures and Tables

**Table 1:** Computerised tomography (CT) and magnetic resonance imaging (MRI) scans of brain.

Date	CT Scan of Brain	MRI Scan of Brain
July 2011	Residual tumour	
January 2012		Stable
May 2012		Stable
September 2012	Reduced tumour size	
August 2013	Stable	
October 2013		Stable
March 2014		Progression of brain tumour with new and increasing foci
June 2014		Likely disease progression
September 2014		Increase size of brain tumour over 1 year, 4 discrete lesions

**Table 2:** Health-related quality of life following intravenous vitamin C (IVC) treatments.

Scales	Before IVC	3 Months after IVC	12 Months after IVC
Symptoms scales			
Fatigue	100	0	0
Nausea	0	0	0
Pain	0	0	0
Dyspnoea	100	0	0
Insomnia	100	0	0
Appetite loss	100	0	0
Constipation	0	0	0
Diarrhoea	67	0	0
Functional scales			
Physical functioning	27	100	100
Role functioning	0	100	100
Emotional functioning	100	100	100
Cognitive functioning	100	100	100
Social Functioning	100	100	100
Global health status	0	100	83
